# Temporal changes in soil microbial properties and nutrient dynamics under climate smart agriculture practices

**DOI:** 10.1016/j.still.2020.104595

**Published:** 2020-05

**Authors:** H.S. Jat, Madhu Choudhary, Ashim Datta, A.K. Yadav, M.D. Meena, Ritu Devi, M.K. Gathala, M.L. Jat, A. McDonald, P.C. Sharma

**Affiliations:** a ICAR-Central Soil Salinity Research Institute (CSSRI), Karnal, 132001, Haryana, India; b International Maize and Wheat Improvement Center (CIMMYT), New Delhi, 110012, India; c International Maize and Wheat Improvement Center (CIMMYT), Dhaka, Bangladesh; d Collage of Agriculture and Life Sciences, Corenell University, Ithaca, NY 14853

**Keywords:** Microbial biomass carbon and nitrogen, Alkaline phosphatase, Dehydrogenase*β*-Glucosidase, Nutrient uptake, Wheat yield

## Abstract

Climate smart agriculture (CSA) practices are emerging as sustainable alternative to conventional rice-wheat system to pull up natural resources degradation across south Asia. After five years of continuous CSA based experiment, a two years study was conducted to evaluate changes in microbial biomasses (microbial biomass carbon and nitrogen), enzyme activities (alkaline phosphatase, dehydrogenase and *β*-glucosidase), nutrient release and uptake (N, P and K) at different wheat crop growth stages. Effect of CSA practices was also studied for carbon mineralization in an incubation experiment. Four scenarios (Sc) were included in this study- conventional tillage (CT) based rice-wheat system (Sc1), partial CSA based rice-wheat-mungbean system (Sc2), full CSA based rice-wheat-mungbean system (Sc3), and full CSA based maize-wheat-mungbean system (Sc4). Soil samples were collected from scenarios at 0–15 and 15−30 cm depth at different growth stages of wheat crop namely sowing, crown root initiation (CRI), active tillering, panicle initiation, and harvesting. Analysis of soil was done for chemical properties *viz*. pH, electrical conductivity, available N, P, K, NPK uptake and mineralizable carbon and biological properties *viz*., microbial biomass carbon (MBC), microbial biomass nitrogen (MBN), dehydrogenase activity (DHA), alkaline phosphatase activity (APA) and β-glucosidase. Significantly higher microbial biomass carbon (42 %) and nitrogen (79 %) were found in surface soil (0−15 cm depth) under CSA based scenarios (Sc2, Sc3 and Sc4) at harvest stage of wheat over CT based/ conventional scenario (Sc1). At surface soil, alkaline phosphatase, dehydrogenase and *β*-glucosidase activity was 58, 14 and 13 % higher in CSA based scenarios, respectively than CT based scenario. CSA based scenarios showed significantly higher C mineralization after 3 days of the incubation experiment at harvest. An increase of respectively 15, 48 and 17 % of N, P and K uptake was observed with CSA based scenarios than CT based scenario. At harvest stage, 7 % higher amount of dry matter was reported with full CSA based scenarios (mean of Sc2 to Sc4) compared to Sc1. Higher wheat grain yield of ∼10 % was recorded with CSA based scenarios over CT based scenario. Therefore, CSA based scenarios with improved biological properties and nutrient availability and uptake at different wheat growth stages resulted in higher yields and hence need to be popularized among the farmers.

## Highlights

• Higher soil microbial biomass carbon (MBC) and nitrogen (MBN) was found under Climate Smart Agriculture (CSA) practices.
• Extracellular enzyme activities were higher at tillering and panicle initiation stage of wheat plant growth
• MBC, MBN and enzyme activity varied with wheat growth stages
• Nutrient uptake,C mineralization and wheat grain yield were higher in CSA based scenarios.

## 1 Introduction

Rice-wheat is the dominating cropping system in Indo-Gangetic plains (IGP) of South Asia occupying nearly 13.5 million hectares area (Gupta and Seth, 2007). Sustainability of this important cropping system is at peril because of intensive tillage based management practices and open field crop residue burning that resulted in natural resources (soil, ground water, energy) degradation. Due to over-exploitation of natural resources, system productivity of rice-wheat is plateauing in IGP (Ladha et al., 2003). The natural resources in IGP are several times more stressed due to population and political pressure compared to rest of the world (Jat et al., 2017). Climate-Smart Agriculture (CSA) is an approach that sustainably increases crop productivity, system resilience (adaptation), reduces the GHGs emission, and enhances achievement of national food security and development goals (FAO, 2013; Lipper et al., 2014). CSA based management practices are emerging as an alternative to reverse the process of natural resource degradation and to maintain the systems sustainability (Govaerts et al., 2009; Hobbs et al., 2007). CSA is based on the concept of conservation agriculture (CA) which involves zero-tillage, crop residue retention; precise water and nutrient management along with efficient crop rotation. In CSA, crop production deals with the management of available agricultural resources with latest management practices and farm machinery under a particular set of edaphic and environmental conditions.

CSA based management practices in isolation may or may not play their potential role in adapting to climate risks in RW system. Therefore, suitable combinations of these management practices may help in building resilience to extreme climate variability to ensure future food security in the region. In IGP, CA based crop management practices significantly alters soil physical, chemical and biological properties (Jat et al., 2017), which lead to alteration in the composition and distribution of soil microbial communities (Choudhary et al., 2018c, d). Interactions between the soil microbial communities and soil organic matter have important role in driving soil functions in any agro-ecosystem, and understanding of this interaction can be supported by soil microbial biomass concept (Stockdale and Murphy, 2017). Soil microbial biomasses help in regulating nutrients like carbon (C) and nitrogen (N) through the process of immobilization and mineralization O’donnell et al. (2001) and considered as sensitive indicators towards crop management practices (Benintende et al., 2008; Gosai et al., 2010).

Changes in microbial dynamics can be reflected by differences in enzyme activities in soils (Dick and Kandeler, 2005). Soil enzymes are important to catalyze many important reactions necessary for decomposition of organic waste, soil structure stabilization, formation of organic matter and nutrient cycling (Dick et al., 1994, 1996). Soil enzyme activity assay is a way to measure the status of soils in the ecosystem (Utobo and Tewari, 2015). Soil enzymes have been reported as important soil quality indicators in any production system, due to their relationship with soil biology and are described as "biological fingerprints" of past soil management (Bandick and Dick, 1999). Soil enzymes are constantly being synthesized, accumulated, inactivated, and decomposed in the soil, hence play a vital role in nutrients cycling (Karaca et al., 2010; Tabatabai, 1994). These are very sensitive to the changes in the soil environment due to different crop management practices like tillage, cropping system, residue and nutrient management (Mangalassery et al., 2015; Balota et al., 2004; Acosta-Martínez et al. (2003); Purakayastha et al., 2007; Lehman et al., 2015; Choudhary et al., 2018a, Choudhary et al., 2018). β-glucosidase is involved in the enzymatic degradation of cellulose by hydrolyzing various β-glucosides present in plant debris (Ajwa and Tabatabai, 1994; Alef and Nannipieri, 1995). β-glucosidase give reflection of past biological activity, soil stabilization capacity, and thus can be used to perceive crop management effect on soils (Bandick and Dick, 1999; Ndiaye et al., 2000; Choudhary et al., 2018a, Choudhary et al., 2018). Phosphatases play important roles in the bioavailability of organic P (Speir and Ross, 1978) and these are affected by different system management practices (Wang et al., 2011; Zhang et al., 2010). DHA activity is considered as an important indicator of soil health and quality (Bera et al., 2017), it also reflects the real picture of overall soil microbial activities. Because of dehydrogenase activity present only in viable cells; it is thought to reflect the total range of oxidative activity of soil microflora and consequently are considered as a good indicator of microbial activity (Nannipieri et al., 1990).

Nutrient release and uptake by plants is facilitated by soil microbial activity and decomposition of crop residues. Decomposition of crop residues under CSA practices further enhance C mineralization and make nutrients available to plants (Datta et al., 2019). Therefore, soil biological properties, carbon mineralization and simultaneously nutrient release and their uptake by crop at different wheat growth stages are strongly related.

The relationship between grain yield and crop biomass accumulation can help in accomplishing improvements in yield through better agronomic management practices and plant breeding (Slafer et al., 1999; Malhi et al., 2006). Grain yield usually increases with simultaneous increase in total dry biomass and nutrient uptake under optimum growing conditions (Karlen and Camp, 1982; Malhi et al., 2006). Distribution patterns of biomass accumulation within plant, its amount, dynamics and nutrient uptake differ with crop growth stages (Karlen and Whitney, 1980), and are influenced by crop cultivars and climatic conditions (Gawronska and Nalborczyk, 1989). Therefore it is required to determine the total nutrient availability and the temporal pattern of their uptake at different crop growth stages for synchronization of nutrient supply with crop nutrient demand to optimise fertilizer recommendations (Abdin et al., 1996; Malhi et al., 2006).

Earlier studies have been done in IGP on effect of CA practices on soil chemical, physical and biological properties were either after harvesting of crop (Jat et al., 2017, 2019; Choudhary et al., 2018a, Choudhary et al., 2018) or with different crop rotations (Parihar et al., 2018). Paucity of literature is available on the temporal changes in soil biological parameters, nutrient availability and mineralizable carbon (C) with crop growth stages under CSA practices. It is necessary to know the effects of CSA based management practices on nutrient release and uptake of wheat crop to ensure balanced nutrition during the particular crop growth stages. This study was carried out with the hypothesis that during different critical crop growth stages whether soil biological properties as well as nutrient release and uptake vary with different CSA based management practices or not. With this background, the objectives of the present study was to evaluate (1) the effect of CSA practices on extracellular soil enzyme activities and microbial biomasses over conventional practice and (2) releasing pattern of nitrogen (N), phosphorus (P) and potassium (K) and their uptake and mineralizable C at different growth stages of wheat.

## 2 Materials and methods

### 2.1 Study site characteristics

A field experiment was set up in 2009 on the experimental farm of Indian Council of Agricultural Research - Central Soil Salinity Research Institute (29°70′N, 76°95′E), Karnal, Haryana, India (Gathala et al., 2013). Climate of the region is semi-arid and sub-tropical with extreme hot and dry (April- June) to wet summers (July-September) and cold dry winters (October-March), with an average annual rainfall of 670 mm, 75–80% of which is received during south-west monsoon. The soil of the experimental field is loam in texture. It falls under *Typic Natrustalf* category (Soil Survey Division Staff, 1993).

### 2.2 Experimental scenarios

The experiment included four cereal-based scenarios varying in cropping system, tillage, crop establishment methods, and residue management practices. Treatments were replicated thrice in 20 m × 100 m plot size in randomized complete block design. The scenarios were designed keeping in view of present practices as well as future drivers of agricultural changes in the region and their details can be obtained from Gathala et al. (2013). Briefly, scenario 1 (Sc1; business-as-usual; farmers’ practice) is conventional till rice-wheat based system, crop residue were removed, rice by manual transplanting of seedlings in puddled soil and wheat by manual broadcasting. In scenario 2 (Sc2; partial CSA- rice-wheat-mungbean based system) transplanting of rice was done in puddled soil with residue incorporation, wheat and mungbean were by drill seeding in zero-till (ZT) conditions with residue retention. Scenario 3 (Sc3; full CSA- based rice-wheat-mungbean system), crops were sown under ZT condition and crops residue were retained on soil surface and in scenario 4 (Sc4; CSA-based maize-wheat-mungbean system), all the three crops were drill seeded under ZT with residue retention. In four years (2009–2013) a total of 47.9, 56.15, and 65.8 Mg ha^−1^ of crop residues, were added respectively in scenario 2, 3, and 4 (Jat et al., 2019). Residue load of rice varied from 4.1–10.6 Mg ha^-1^, of wheat from 0.7 to 3.6 Mg ha^-1^ and of maize from 9.5–13.7 Mg ha^-1^ under different treatments. Residue of mungbean (2.5 to 4.7 t ha^-1^) was incorporated during puddling operation of rice in Sc2, however, in Sc3 and Sc4 mungbean residues were retained on the soil surface.

### 2.3 Soil sampling and analysis

Soil samples were collected from each plot at 0–15 and 15−30 cm depth by an auger with 5 cm diameter at critical crop growth stages like sowing, crown root initiation (CRI), tillering, panicle initiation, and harvesting of wheat crop during 2014-15 and 2015-16. Samples were collected from nine locations within each plot and a composite sample was prepared by mixing them. Part of the fresh soil samples were kept in a refrigerator at 4 °C for analysis of soil biological parameters. *viz*., MBC, MBN, dehydrogenase activity (DHA), alkaline phosphatase activity (APA) and β-glucosidase activity. The remaining portion of the soil samples were air-dried in shade, ground to pass through a 2-mm sieve, stored in plastic jar for analysis of soil chemical properties *viz*. pH, EC, available N, P, K. Soil pH and electrical conductivity (EC) in soil: water ratio of 1:2 was determined by following standard methods (Jackson, 1973). MBC and MBN were estimated by chloroform fumigation method (Vance et al., 1987). Dehydrogenase and alkaline phosphatase activities were estimated as described by Dick et al. (1996). β-glucosidase activity was determined by the method of Eivazi and Tabatabai (1988). Mineralizable carbon was estimated by CO_2_–C evolution method with fresh soil samples (Anderson, 1982). In brief, the amount of CO_2_ evolved, during the incubation period (23-day), was absorbed in 10 mL of 0.5 *N* NaOH solution and then titrated with 0.5 *N* HCl using phenolphthalein indicator. The available nitrogen (N) in soil was determined by alkaline permanganate method (Subbiah and Asija, 1956). Available phosphorus (Olsen- P) in soil was determined colorimetrically following ascorbic acid reductant method as outlined by Olsen et al. (1954). Available potassium (K) in soil was determined by flame photometer using neutral 1*N* ammonium acetate extractant as described by Jackson (1973). N, P and K uptake was calculated by analysing plant N, P and K concentration following standard method (Jackson, 1973).

### 2.4 Dry matter accumulation and grain yield

The dry matter accumulation (DMA) was recorded at different growth stages of wheat crop (Ihsan et al., 2016). Crop samples were collected from 1 m × 1 m area for DMA at different crop stage. The crop samples were oven dried and weighted. The wheat crop was harvested manually from 4 m × 4 m randomly selected three places from each plot for recording the grain yield. Grain yield was reported at 12 % moisture.

### 2.5 Statistical analysis

The data were subjected to analysis of variance (ANOVA) and using the general linear model procedure of the SPSS window version 17.0 (SPSS Inc., 1999). Treatment means were separated by Duncan Multiple Range Test at 5 % level of significance (*P<*0.05) Table 1.

**Table 1:** Drivers of change and management practices in different scenarios.

Scenarios (Sc)	Sc1-Conventional tillage (CT) based		Sc2- Partial climate smart agriculture (CSA) based			Sc3- Full CSA based			Sc4- Full CSA based		
Drivers of Change	Conventional practice/ Business as usual (Farmer’s Practice)		Increase productivity and profitability through intensification and best management practices			To deal with rising scarcity of water, energy, labor, and expected climate change			Futuristic cropping system to deal with same issues as in Sc3		
Crop Rotations	Rice-Wheat- Fallow		Rice-Wheat-Mungbean			Rice-Wheat-Mungbean			Maize-Wheat- Mungbean		
	Rice	Wheat	Rice	Wheat	Mungbean	Rice	Wheat	Mungbean	Maize	Wheat	Mungbean
Tillage	CT	CT	CT	Zero tillage (ZT)	ZT	ZT	ZT	ZT	ZT	ZT	ZT
Crop Establishment Method	Transplanting	Broadcasting	Transplanting	Drill seeding	Drill/relay	Drill seeding	Drill seeding	Drill/relay	Drill seeding	Drill seeding	Drill/relay
Residue Management	Removed	Removed	Retained full (100 %)	Retained anchored (30 %)	Incorporated full (100 %)	Retained full (100 %)	Retained anchored (30 %)	Retained full (100 %)	Retained partial (65 %)	Retained anchored (30 %)	Retained full (100 %)
Nutrient Management (NPK, kg/ ha)	175 + 58 + 0	150 + 58 + 0	151 + 58 + 60	151 + 64 + 32	0 + 0+0	162 + 64 + 62	151 + 64 + 32	0 + 0+0	174 + 64 + 62	151 + 64 + 32	0 + 0+0
Water Management	Continuous flooding of 5-cm depth for 1 month, followed by irrigation applied at hair-line crack	Need based irrigation or at critical crop growth stages	Continuous flooding of 5-cm depth for first 15–20 days after transplanting ‘fb’ irrigation at -40 to −50 kPa matric potential at 15-cm depth till 1 wk before flowering ‘fb’ irrigation at -15 to -20 kPa	Flood irrigation at -40 to−50 kPa matric potential	Flood irrigation as and when required	Kept soil wet for first 20 days ‘fb’ irrigation at -20 to −30 kPa matric potential	Flood irrigation at -40 to−50 kPa matric potential	Flood irrigation as and when required	Flood Irrigation at −50 kPa in maize and	Flood irrigation at -40 to−50 kPa matric potential	Flood irrigation as and when required

## 3 Results

### 3.1 Microbial biomass carbon (MBC) and nitrogen (MBN)

Data pertaining to microbial biomasses (MBC and MBN) at different crop growth stages are presented in Table 2, Table 3. With some exceptions, MBC was significantly higher at surface and sub-surface soil in Sc4 compared to other scenarios. However, lower values were recorded in sub-surface soil (15−30 cm) compared to surface soil (0−15 cm). At harvest stage of wheat MBC was in order of Sc4 (210 μg g^−1^ dry soil) >Sc3 (187.6 μg g^-1^dry soil) >Sc2 (171.7 μg g^-1^dry soil) >Sc1 (133.3 μg g^-1^dry soil) and was significantly higher over one another (Table 2). At sowing of wheat, MBC was significantly higher in Sc2 and Sc3 as compared to Sc1 and Sc4. Trend of MBC was increased from sowing to harvest stage of wheat with a decrease at tillering stage in both the soil depths. In sub-surface soil depth, MBC was significantly higher in Sc4 at all growth stages of wheat over other scenarios. Higher concentration of MBC had registered with Sc4 (188.8 μg g^-1^dry soil) and lowest with Sc1 (82.6 μg g^-1^dry soil) in sub-surface soil layer at harvest of wheat (Table 2).

**Table 2:** Effect of different CSA-based scenarios on MBC (μg g^−1^ dry soil) during wheat crop growth stages (2 yrs’ mean).

Scenarios^a^	Sowing	Crown Root Initiation	Tillering	Panicle initiation	Harvest
Surface soil (0−15 cm depth)					
1	29.9C^b^	65.8C	44.8C	73.3C	133.3D
2	56.6A	77.1AB	60.6B	115.3B	171.7C
3	52.1A	74.2B	67.9A	123.2A	187.6B
4	42.9B	81.7A	66.4A	126.8A	210.0A
Sub-surface soil (15−30 cm depth)					
1	19.2B	44.2C	29.1B	58.6C	82.6C
2	37.7A	55.8B	34.8B	99.5B	143.6B
3	37.5A	50.0BC	34.6B	117.9A	171.2A
4	39.7A	68.9A	51.1A	113.5A	188.8A

	Main effects	*P- value*
	Scenario (S)	<0.05
	Depth (D)	<0.05
	Growth stage (GS)	<0.05
	Year (Y)	NS
	Interaction effects	
	GS × S	<0.05
	S × Y	<0.05
	GS × Y	<0.05

*Where*; CSA- climate smart agriculture, MBC- Microbial biomass carbon, Sc1: conventional rice-wheat system, Sc2: partial CSA based rice-wheat-mungbean system, Sc3: full CSA based rice-wheat-mungbean system, Sc4: full CSA based maize-wheat-mungbean system.

a Refer Table 1 for scenario description.

b Means followed by a similar uppercase letters within each column are not statistically different (P ≤ 0.05, Duncan’s multiple range test).

**Table 3:** Effect of different CSA-based scenarios on MBN (μg g^−1^ dry soil) during wheat crop growth stages (2 yrs’ mean).

Scenarios^a^	Sowing	Crown Root Initiation	Tillering	Panicle initiation	Harvest
Surface soil (0−15 cm depth)					
1	4.3B^b^	6.0C	4.7C	9.2C	11.1C
2	5.7A	12.9A	7.2B	12.8B	19.1B
3	6.1A	8.7B	7.6B	14.5AB	17.1B
4	4.5B	9.1B	11.1A	15.9A	23.3A
Sub-surface soil (15−30 cm depth)					
1	2.3B	4.9B	3.6B	6.9C	8.3C
2	4.2AB	5.6B	3.9B	10.5B	16.9A
3	3.8AB	5.3B	3.1B	12.2A	12.2B
4	5.7A	6.9A	5.7A	8.7C	13.5B

	Main effects	*P- value*
	Scenario (S)	NS
	Depth (D)	<0.05
	Growth stage (GS)	<0.05
	Year (Y)	NS
	Interaction effects	
	GS × S	<0.05
	S × Y	<0.05
	GS × Y	<0.05

*Where*; CSA- climate smart agriculture, MBC- Microbial biomass nitrogen, Sc1: conventional rice-wheat system, Sc2: partial CSA based rice-wheat-mungbean system, Sc3: full CSA based rice-wheat-mungbean system, Sc4: full CSA based maize-wheat-mungbean system.

a Refer Table 1 for scenario description.

b Means followed by a similar uppercase letters within each column are not statistically different (P ≤ 0.05, Duncan’s multiple range test).

In general MBN was found lower in sub-surface soil depth as compared to surface soil in all scenarios. Higher MBN was recorded from tillering to harvesting stage with Sc4 compared to other scenarios, whereas Sc2 performed better at sowing and CRI stage. Similar to MBC, MBN was also significantly higher in surface soil with Sc4 (23.3 μg g^−1^dry soil) followed by Sc2 (19.1 μg g^−1^dry soil), Sc3 (17.1 μg g^−1^dry soil) and Sc1 (11.1 μg g^−1^dry soil) (Table 3). MBN was increased from sowing to harvest with exceptions at tillering stage. Over all MBN was higher at harvest stage of wheat in comparison to other stages of crop growth irrespective of scenarios. In sub-surface soil depth (15−30 cm), there were nonsignificant differences reported in MBN within sowing to tillering stages of wheat among Sc1 to Sc3 (Table 3). Sc4 had higher value of MBN irrespective of growth stages of wheat in sub-surface soil. At harvest stage, MBN was 62.6 % higher in Sc4 over Sc1. Overall, higher MBC and MBN were observed in partial (Sc2) and full CSA based scenarios (Sc3and Sc4) at both the depths as compared to CT based scenario (Sc1). At surface soil depth, MBC and MBN were 42 % and 79 % higher in partial and full CSA based scenarios, respectively than CT based scenario at harvesting stage. Interaction effect of growth stage × scenario, scenario × year and growth stage × growth stage on MBC and MBN were found significant.

### 3.2 Extracellular enzyme activities

At the surface soil (0−15 cm) highest APA (148.28 μg p-NP g^−1^ soil h^−1^) was recorded with Sc3 at sowing stage of wheat however; decrement was observed towards maturity of crop in all scenarios (Table 4). Lowest values of APA were reported with Sc1 except at sowing stage and highest being associated with either Sc2 or Sc3 at different growth stages. Similar trends were also reported in sub-surface soil in all scenarios. Maximum APA was reported at sowing stage as compared to other stages of wheat except tillering in Sc3. At harvest, significantly higher amount of APA was reported in Sc2 (98.03 μg g^−1^ soil h^−1^) than Sc1, however it was statistically similar to Sc3 and Sc4. Higher and lower values of APA were, respectively reported with sowing and CRI stages than other growth stages at both soil depths. At harvest, under CSA based scenarios (mean of Sc2 to Sc4) APA was 58 % and 31 % higher in surface and sub-surface soil, respectively than CT based scenario (Sc1).

**Table 4:** Effect of different CSA-based scenarios on alkaline phosphatase (μg p-NP per g soil per hr) during wheat crop growth stages (2 yrs’ mean).

Scenarios^a^	Sowing	Crown Root Initiation	Tillering	Panicle initiation	Harvest
Surface soil (0−15 cm depth)					
1	104.62C^b^	67.41D	78.78D	81.55D	73.23C
2	98.54C	89.95A	139.97A	119.08B	122.95A
3	148.28A	82.46B	107.32B	131.80A	115.11AB
4	122.21B	75.89C	94.94C	102.95C	108.79B
Sub-surface soil (15−30 cm depth)					
1	97.26B	47.91B	74.81B	62.12C	71.92B
2	153.42A	63.19A	110.95A	100.58A	98.03A
3	115.26B	58.56A	116.06A	89.49B	94.38A
4	100.04B	57.62A	78.70B	85.89B	89.20A

	Main effects	*P- value*
	Scenario (S)	<0.05
	Depth (D)	<0.05
	Growth stage (GS)	<0.05
	Year (Y)	<0.05
	Interaction effects	
	GS × S	NS
	S × Y	<0.05
	GS × Y	<0.05

*Where*; CSA- climate smart agriculture, Sc1: conventional rice-wheat system, Sc2: partial CSA based rice-wheat-mungbean system, Sc3: full CSA based rice-wheat-mungbean system, Sc4: full CSA based maize-wheat-mungbean system.

a Refer Table 1 for scenario description.

b Means followed by a similar uppercase letters within each column are not statistically different (P ≤ 0.05, Duncan’s multiple range test).

DHA had significantly higher values with CSA based scenarios at all growth stages except sowing stage as compared to Sc1 at surface soil (Table 5). DHA was remarkably increased up to tillering stage and then decreased in all scenarios. However, highest amount of DHA was observed at tillering stage of Sc2 (117.87*μg* TFT g soil^−1^ 24 h^−1^) and Sc4 (116.33 *μg* TFT g soil^−1^ 24 h^−1^). Similar to surface soil, sub-surface depths also showed similar pattern (Table 5). In all growth stages, higher amount of DHA was observed with Sc2 than other scenarios. Similar to surface soil, it was highest at tillering stage of Sc2 (66.81*μg* TFT g soil^−1^ 24 h^−1^).

**Table 5:** Effect of different CSA-based scenarios on Dehydrogenase activity (μg TPF /g soil/24 h) during wheat crop growth stages (2 yrs’ mean).

Scenarios^a^	Sowing	Crown Root Initiation	Tillering	Panicle initiation	Harvest
Surface soil (0−15 cm depth)					
1	92.69A^b^	47.56D	99.02B	98.77B	69.50B
2	60.90B	61.36C	117.87A	114.48A	84.72A
3	71.54B	77.56B	109.95AB	104.59AB	83.52A
4	70.75B	102.62A	116.33A	111.35A	69.11B
Sub-surface soil (15−30 cm depth)					
1	33.51B	30.59C	39.23C	35.86B	38.40C
2	60.35A	54.04A	66.81A	44.34A	56.05A
3	39.49B	47.48AB	54.02B	48.96A	49.73AB
4	33.04B	38.96BC	41.99C	47.09A	44.08BC

	Main effects	*P- value*
	Scenario (S)	<0.05
	Depth (D)	<0.05
	Growth stage (GS)	<0.05
	Year (Y)	<0.05
	Interaction effects	
	GS × S	<0.05
	S × Y	<0.05
	GS × Y	<0.05

*Where*; CSA- climate smart agriculture, Sc1: conventional rice-wheat system, Sc2: partial CSA based rice-wheat-mungbean system, Sc3: full CSA based rice-wheat-mungbean system, Sc4: full CSA based maize-wheat-mungbean system.

a Refer Table 1 for scenario description.

b Means followed by a similar uppercase letters within each column are not statistically different (P ≤ 0.05, Duncan’s multiple range test).

**Fig. 1:**
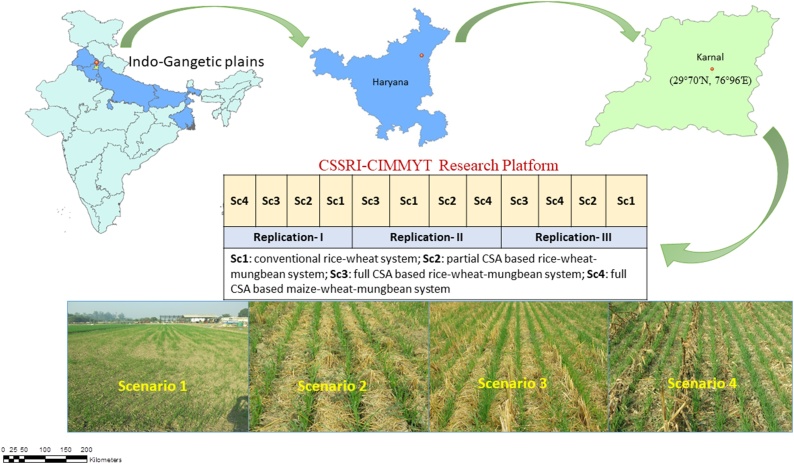
Location and layout of experiment.

CSA based scenarios (Sc2, Sc3 and Sc4) resulted significant change in *β*-glucosidase activity at different growth stages as compared to CT based scenario/ Sc1 (Table 6). *β*-glucosidase was gradually increased from sowing to panicle initiation (PI) in all scenarios. However, maximum value of *β*-glucosidase was reported at PI stage with Sc4 (79.05 μg g^−1^ soil h^−1^) at surface soil depth which is statistically significant over the other scenarios. Similar to surface soil, *β*-glucosidase activity was increased from sowing to PI stage of wheat at subsurface soil depth (Table 6). At PI, CSA based scenarios had 43 % and 68 % higher *β*-glucosidase activity at surface and sub-surface soil depths, respectively, over Sc1. At harvest, *β*-glucosidase activity was lower than PI but it was 13 % and 28 % higher in CSA based scenarios than CT based scenario. All soil enzyme activities were found to be higher in partial CSA and full CSA based scenarios as compared to CT based scenario at both the soil depths Fig. 1.

**Table 6:** Effect of different CSA-based scenarios on *β*-Glucosidase (μg p-NP per g soil per hr) during wheat crop growth stages (2 yrs’ mean).

Scenarios^a^	Sowing	Crown Root Initiation	Tillering	Panicle initiation	Harvest
Surface soil (0−15 cm depth)					
1	5.70B^b^	5.38B	8.84C	44.40C	34.50B
2	10.63A	9.22A	15.80A	49.65C	36.30B
3	9.62A	8.26A	9.48C	61.08B	36.04B
4	9.59A	9.73A	12.09B	79.05A	44.59A
Sub-surface soil (15−30 cm depth)					
1	3.77C	4.48C	5.05B	33.32B	26.26B
2	6.01A	5.35B	5.68B	43.24B	35.09A
3	4.65B	4.98BC	5.89B	61.98A	33.46A
4	4.58B	6.70A	7.28A	63.03A	31.93A
					

	Main effects	*P- value*
	Scenario (S)	<0.05
	Depth (D)	<0.05
	Growth stage (GS)	<0.05
	Year (Y)	NS
	Interaction effects	
	GS × S	<0.05
	S × Y	NS
	GS × Y	<0.05

*Where*; CSA- climate smart agriculture, Sc1: conventional rice-wheat system, Sc2: partial CSA based rice-wheat-mungbean system, Sc3: full CSA based rice-wheat-mungbean system, Sc4: full CSA based maize-wheat-mungbean system.

a Refer Table 1 for scenario description.

b Means followed by a similar uppercase letters within each column are not statistically different (P ≤ 0.05, Duncan’s multiple range test).

### 3.3 Carbon (C) mineralization

**Fig. 2:**
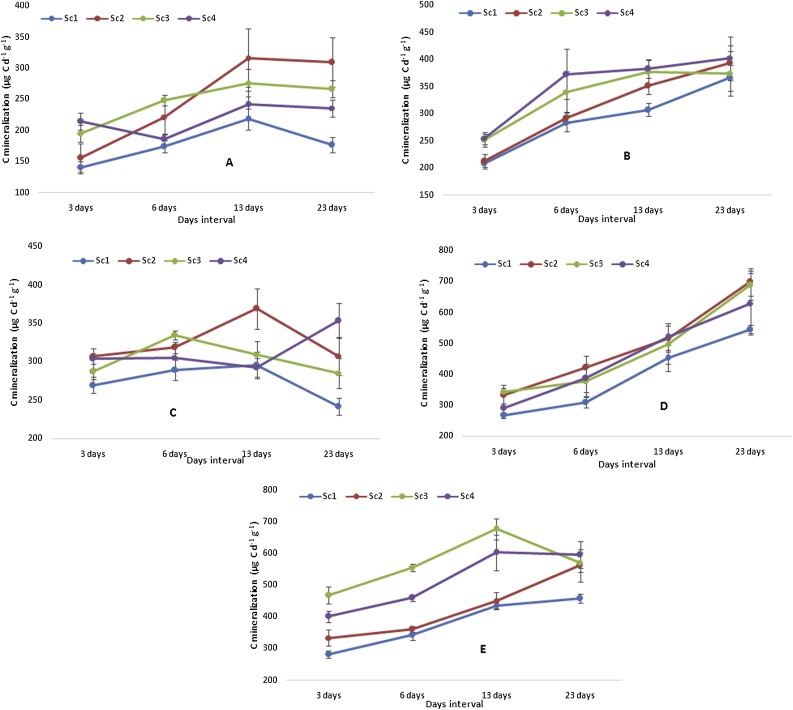
Variation in mean mineralizable carbon at different growth stages of wheat (2 yrs’ mean) A) sowing B) crown root initiation C) tillering D) Panicle initiation E) Harvest. Vertical bars indicate ± Standard error of mean.

Carbon mineralization significantly varied among the scenarios at different growth stages of wheat (Fig. 2). At sowing, highest C mineralization was observed under Sc2 (317 μg d^−1^ g^−1^ of dry soil) and Sc3 (276 μg d^−1^ g^−1^ of dry soil) at 13 days of the incubation and lowest was associated with Sc1 (218 μg d^−1^ g^−1^ of dry soil). Similar trend was also observed at 23 day’s interval. At 3 and 6 days interval Sc4 and Sc3 recorded higher C mineralization, respectively than other scenarios. At CRI stage, as the time progresses irrespective of scenarios C mineralization increased and highest was observed under Sc4 (403 μg d^−1^ g^−1^ of dry soil) followed by Sc2 (393 μg d^−1^ g^−1^ of dry soil) after 23 days of incubation (Fig. 2). At tillering stage, interesting observation was recorded. Initially at 3 days interval, higher C mineralization was observed in all the scenarios but after 6 days interval significant decline was observed irrespective of scenarios except Sc2. After 23 days, highest C mineralization was observed in Sc4 (353 μg d^−1^ g^−1^ of dry soil). Irrespective of interval days significantly higher C mineralization was observed in panicle initiation stage than at sowing, CRI and tillering stage. Highest C mineralization was recorded in Sc3 (732 μg d^−1^ g^−1^ of dry soil) and Sc2 (722 μg d^−1^ g^−1^ of dry soil) after 23 days of incubation. At harvest, CSA based scenarios showed significantly higher C mineralization after 3 days of the incubation experiment. In Sc3 and Sc4, after 13 days, there was decline in C mineralization and highest was associated with Sc4 (623 μg d^−1^ g^−1^ of dry soil) and Sc2 (624 μg d^−1^ g^−1^ of dry soil) after 23 days of the experiment (Fig. 2).

### 3.4 Changes in nutrient availability and their uptake

**Fig. 3:**
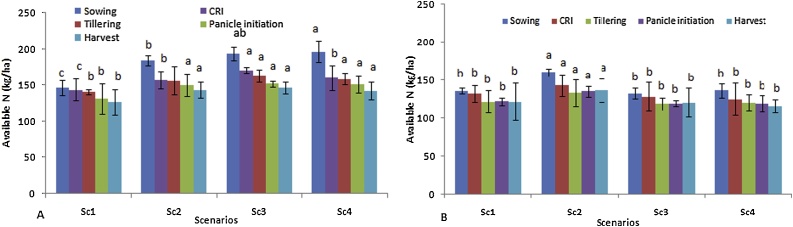
Effect of different CSA-based scenarios on available N (kg ha^−1^); A) at 0−15 cm soil depth and B) at 15−30 cm soil depth during wheat crop growth stages (2 yrs’ mean). Where CSA- climate smart agriculture, N- Nitrogen. Means followed by a similar lower case letters within each column are not statistically different (P ≤ 0.05, Duncan’s multiple range tests). Vertical bars indicate ± Standard error of mean.

**Fig. 4:**
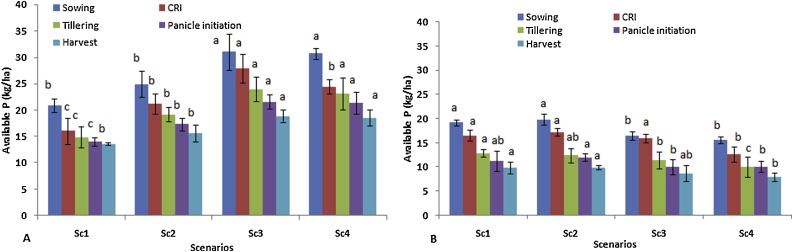
Effect of different CSA-based scenarios on available P (kg ha^−1^); A) at 0−15 cm soil depth and B) at 15−30 cm soil depth during wheat crop growth stages (2 yrs’ mean). Where CSA- climate smart agriculture, P- Phosphorus. Means followed by a similar lower case letters within each column are not statistically different (P ≤ 0.05, Duncan’s multiple range tests). Vertical bars indicate ± Standard error of mean.

Nitrogen (N) availability at surface soil was reduced from sowing to harvest stage of wheat irrespective of scenarios (Fig. 3). Significantly higher N availability was observed in Sc4 (196 kg N ha^−1^) at sowing of wheat over all other scenarios. The maximum reduction in N availability was observed in Sc4 (27.5 %) from sowing to harvest stage whereas, minimum was observed in Sc1 (13.65 %). Similar trends of N reduction were reported in sub-surface soil depth in all scenarios from sowing to harvesting (Fig. 3). In sub-surface depth, available N was higher in Sc2 than other scenarios at all growth stages. However, lower availability of N was observed in sub-surface as compared to surface soil in all scenarios. Similar to available N, available P was also significantly reduced in all scenarios from sowing to harvest stage in surface soil depth (Fig. 4) and ranged from 35 to 40 %. Significantly higher available P was found in full CSA based scenarios compared to Sc1 and Sc2 at all growth stages. In sub-surface soil, pattern of P availability was also similar to surface soil in all scenarios (Fig. 4). Similar to results of available N, P was also comparatively higher in Sc2 than Sc3 and Sc4.

**Fig. 5:**
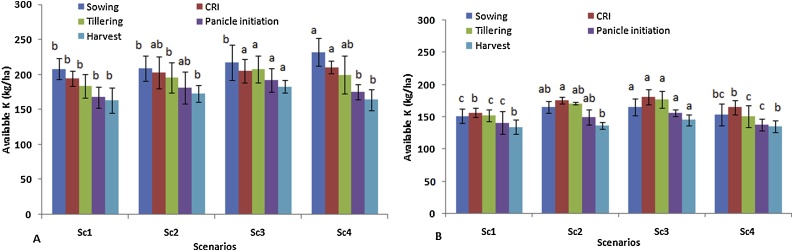
Effect of different CSA-based scenarios on available K (kg ha^−1^); A) at 0−15 cm soil depth and B) at 15−30 cm soil depth during wheat crop growth stages (2 yrs’ mean). Where CSA- climate smart agriculture, K- Potassium. Means followed by a similar lower case letters within each column are not statistically different (P ≤ 0.05, Duncan’s multiple range tests). Vertical bars indicate ± Standard error of mean.

Significantly (*p* < 0.05) higher available K (232 kg ha^−1^) was observed in surface soil of Sc4 at sowing stage over other scenarios (Fig. 5). The decreasing trend of K was almost similar to N and P from sowing to harvest stage of wheat in all scenarios. Available K was 16–29 % lower from sowing to harvest with different scenarios, highest decrease (29 %) was observed with Sc4. In sub-surface soil, the availability of K was lower in all scenarios in comparison to surface soil (Fig. 5). Available K was decreasing from sowing to harvest stage of wheat and followed the same trend in case of N and P. At all growth stages higher available K was reported with Sc3. Interaction effect of growth stage × scenario, scenario × year and growth stage × year were found non-significant with available N, P and K except interaction of scenario × year in K availability (Table 7).

**Table 7:** Main and interaction effect of Scenarios, soil depth, growth stages and year for available Nitrogen (N), Phosphorus (P) and Potassium (K).

Main effects	Available N	Available P	Available K
Scenario (S)	**	**	**
Depth (D)	**	**	**
Growth stage (GS)	**	**	**
Year (Y)	**	**	**
Interaction effects			
GS × S	NS	NS	NS
S × Y	NS	NS	**
GS × Y	NS	NS	NS

**indicates significant at 5 % level of significance (p < 0.05).

Uptake of N, P and K was increased from CRI to harvest stage of wheat irrespective of scenarios (Table 8). Significantly higher uptake of N, P and K was observed in full CSA based scenario followed by partial CSA and CT based scenario. An increase of respectively 15, 48 and 17 % of N, P and K uptake was observed with CSA based scenarios than CT based scenario at harvest stage.

**Table 8:** Effect of different CSA-based scenarios on NPK uptake (kg ha^−1^) during wheat crop growth stages (2 yrs’ mean).

Scenarios^a^	Crown Root Initiation	Tillering	Panicle initiation	Harvest
N uptake				
1	1.01B^b^	6.06C	44.52B	109.13B
2	1.26B	7.60BC	49.59A	124.14A
3	2.80A	7.94AB	53.41A	124.21A
4	2.44A	9.65A	54.35A	128.53A
P uptake				
1	0.07B	0.42B	3.97C	15.98C
2	0.10B	0.59AB	4.94B	20.85B
3	0.32A	0.59AB	5.74A	24.50AB
4	0.25A	0.76A	5.48AB	25.69A
K uptake				
1	3.53C	41.33C	117.11C	120.00B
2	4.48C	47.60BC	128.24B	136.85A
3	15.99A	52.25AB	134.50AB	142.64A
4	10.96B	60.53A	137.72A	143.28A

*Where*; CSA- climate smart agriculture, Sc1: conventional rice-wheat system, Sc2: partial CSA based rice-wheat-mungbean system, Sc3: full CSA based rice-wheat-mungbean system, Sc4: full CSA based maize-wheat-mungbean system.

a Refer Table 1 for scenario description.

b Means followed by a similar uppercase letters within each column are not statistically different (P ≤ 0.05, Duncan’s multiple range test).

### 3.5 Dry matter accumulation (DMA) and grain yield

Accumulation of higher dry matter was observed in CSA based scenarios than CT based scenario (Sc1) at different growth stages of wheat (Table 9). Scenario 3 and Sc4 recorded higher and at par DMA at different growth stages of crop. Dry matter accumulation was increased from CRI to harvest stage of wheat. At harvest stage, 7 % higher amount of DM was reported with full CSA based scenarios (mean of Sc2 to Sc4) than CT based scenario (11.21 t ha^−1^). All the CSA based scenarios were at par with respect to grain yield and significantly higher over CT based scenario (5.04 t ha^−1^). Almost 10 % higher gran yield was recorded with CSA based scenario than CT based scenario (Table 9).

**Table 9:** Effect of different CSA-based scenarios on dry matter accumulation (t ha^−1^) at different wheat crop growth stages and grain yield (t ha^−1^) (2 yrs’ mean).

Scenarios^a^	Crown Root Initiation	Tillering	Panicle initiation	Harvest	Yield (t/ha)
1	0.16B^b^	1.25C	8.38B	11.21C	5.04B
2	0.18B	1.52B	8.77B	11.50BC	5.44A
3	0.43A	1.41B	9.26A	12.00A	5.54A
4	0.36A	1.74A	9.37A	11.98AB	5.57A

*Where*; CSA- climate smart agriculture, Sc1: conventional rice-wheat system, Sc2: partial CSA based rice-wheat-mungbean system, Sc3: full CSA based rice-wheat-mungbean system, Sc4: full CSA based maize-wheat-mungbean system.

a Refer Table 1 for scenario description.

b Means followed by a similar uppercase letters within each column are not statistically different (P ≤ 0.05, Duncan’s multiple range test).

## 4 Discussion

### 4.1 Changes in microbial biomass carbon (MBC) and nitrogen (MBN)

Microbial biomasses (MBC and MBN) are considered to be sensitive to agriculture management than changes in total soil organic matter (SOM) (Guo et al., 2013). Climatic factors have less impact on soil microbial biomass than local factors such as plant diversity and soil properties (Thakur et al., 2015). Changes in microbial biomasses are important indicators of soil quality due to the impacts of management practices on soil properties (Choudhary et al., 2018a, Choudhary et al., 2018). In present study, at surface soil depth, MBC and MBN were significantly higher in CSA based scenarios (Sc2, Sc3 and Sc4) than CT based scenario (Sc1), it can be explained that presence of crop residues in CSA based scenarios had created suitable environment for microbial growth (Jat et al., 2019) as crop residue maintains substrate availability, moisture and temperature for better microbial growth (Ghimire et al., 2017). Crop residue acts as a readily available food source and also provides a wide range of nutrients to microbes which resulted in higher microbial biomasses (Masto et al., 2007). Moreover compositional variation of plant biomass (crop residue) also influences soil microbial properties (Li et al., 2018) as different quality and quantity of residue load is present in different scenarios. Microbial biomasses are sensitive towards changes in soil temperature and moisture (Pandey et al., 2015). Residues on the surface act as insulator and maintain soil temperature even in abrupt fluctuations in air temperature (Blanco-Canqui and Lal, 2009). Residues also act as protective cover to reduce evaporation losses and maintain soil moisture (Ghimire et al., 2017). Among different growth stages, higher microbial biomasses were reported at harvest stage of wheat, due to the higher mineralization of crop residues takes place at maturity stage of wheat crop (Fig. 2) (Datta et al., 2019) which provide higher amount of organic matter available for microbial growth (Chandra, 2011). At tillering stage both MBC and MBN were declined which might be due to rapid growth of aerial parts and expansion of roots at this stage which causes more absorbance of nutrient from soil that creates competition with soil microorganism (Zeng et al., 2005). Lower values of MBC and MBN in sub-surface soil irrespective of scenarios in comparison to surface soil is attributed to the presence of higher amount of soil organic matter in surface soil (Meena and Biswas, 2014; Venzke Filho et al. (2004)). At harvest, sub-surface MBN was significantly higher with Sc2 than other scenarios which might be due to residue incorporation facilitating higher mineralization compared to other scenarios where residues were either retained on soil surface and /or removed.

### 4.2 Changes in soil enzyme activities

Agriculture management systems influence microbial activities, as it is evident from extra cellular enzyme activities *viz*. alkaline phosphatase activity (APA), dehydrogenase activity (DHA) and *β*-glucosidase. Overall enzyme activities under CSA based scenarios were higher than conventional practices/ CT based scenario. It is mainly due to minimum disturbance of soils along with residue retention, which provides suitable environment for microbes by moderating soil moisture and temperature than CT based scenario (Choudhary et al., 2018). From sowing to CRI stage APA, DHA and *β*-glucosidase were decreased, and it increased at tillering and PI stages and again decreased at harvest. Decline in enzyme activities at CRI and harvest stages might be due to less efficient root system. At CRI, crown roots/ permanent roots start growing and seminal/ temporary roots got dysfunctional, whereas at harvest root system got dysfunctional. Higher enzyme activities in tillering and PI stages may be ascribed to active and higher root biomass at these stages (Mandal et al., 2007). The reason for the differential behavior of APA is unclear and requires further elucidation.

Results indicated that DHA had higher activity with Sc4 in all stages of wheat crop in comparison to other scenarios, it can be explained that more crop residues were supplied in Sc4 than other scenarios (Jat et al., 2017, 2019). At harvest stage of crop, maximum DHA was reported in all scenarios compared to sowing stage of wheat, it was mainly due to lower mineralization rate of organic materials at sowing stage and it increased toward maturity of wheat in surface soil. In Sc2, at harvest of wheat DHA was 22.7 % higher than Sc4 which might be due to higher amount of substrates (organic matter) available for microbial growth (Chandra, 2011). In sub-surface soil, DHA activity was lower in all stages of crop growth irrespective of scenarios, because microbial activity is decreased with depth.

In our study, it is consistent that CSA practices affected β-glucosidase activity in the surface soil layer. Results indicate that *β-*glucosidase was significantly higher at PI stage of wheat irrespective of scenarios, however in Sc4 (maize based systems) it was 79 % higher than Sc1 (rice based systems) at PI stage. Rabary et al. (2008) indicated that *β-*glucosidase activity in soil is very sensitive to cropping systems and land use management. Also this could be explained by the fact that the decomposition of crop residues supplies biomass carbon input in the form of readily available substrates like carbohydrate, which can increase this enzyme activity (Green et al., 2007). In sub-surface soil *β-*glucosidase activity was lower in all scenarios and crop stages in comparison to surface soil which might be due to lower carbon substrate availability (Jat et al., 2017) to microbes in soil. The highest activities of most of the enzymes at tillering and PI stage of wheat are in agreement with the earlier findings of Bera et al. (2017) who opined that higher enzyme activities are associated with the vigorous vegetative growth stages than the productive growth stages.

### 4.3 Variation in C mineralization

Significant variation in C mineralization among the scenarios at different growth stages of wheat might be due to the effect of crop management practices. Zero tillage and residue retention at soil surface significantly modify the soil environment in terms of regulation of soil temperature, moisture (Coppens et al., 2006; Awad et al., 2012) and microbial activity which might cause the variation in C mineralization. At sowing, in Sc2 higher C mineralization was attributed due to the closer contact between crop residue and soil as the previous crop residues were incorporated during puddling (churning of soil) before rice transplanting which helps well mixing the soil with crop residues. At CRI stage, as the crop root (crown/ permanent) starts growing as well as the decomposition of the crop residues commences, C mineralization increases in CSA based scenarios. In tillering stage, decline in C mineralization during the later part of the incubation might be due to lower microbial activity as during this stage the fast growing microbes of sowing and CRI stages die. But at panicle initiation stage, there might be fresh flush of microbes on the death cells which might cause higher mineralization that resulted in higher N, P and K availability and their uptake in plants. In Sc2, higher C mineralization might be due to the incorporation of crop residue into the soil (Datta et al., 2019). Closer contact between soil and residues incorporated into the soil might be attributed to the faster decomposition of residues in Sc2 (Sims and Frederick, 1970; Bremer et al., 1991; Ambus and Jensen, 1997). Datta et al. (2019) also observed significant (P < 0.001) effect of residue placement on soil. The decomposition rate constant values for surface applied wheat residues were higher than those of the incorporated into the soil which explained higher C mineralization in CSA based scenarios.

### 4.4 Nutrient availability and uptake at different growth stages

Nutrient availability and their uptake by plants were significantly influenced under different CSA based management practices. At sowing, higher available N under CSA based practices was due to the retention of huge (∼14 t ha^−1^ yr^−1^) amount of crop residues at surface soil which upon decomposition supplies N to soil. After four years of the experiment, Jat et al. (2017) also observed higher available N under CA based agricultural practices. With advancement of crop growth stages, available N in soil decreased significantly and higher was observed under CSA based practices which might be due to higher uptake of N (Fig. 3) as compared to conventional practices. Lowest available N at panicle initiation and harvest stage was due to higher crop uptake resulting from higher mineralization of organic matter (Fig. 2) leading to higher availability of N. Mehta et al. (1963) also observed higher N uptake at PI and harvesting stage of wheat. In soil mineralization and immobilization of N takes place simultaneously (Alexander, 1961) resulting in variation of N availability to plants. Datta et al. (2014; Datta and Saha, 2017) also showed variation of available N at different days of application of humic acid and organic matter in soils due to transformation of different organic fractions of N in soil. Devi and Saha (2017) showed the availability of N in soil depends on the transformation of organic and inorganic forms of N in soil upon application of humic acid and organic matters. P and K also showed similar trend. Higher available P and K at sowing was due to higher amount of crop residues retained at soil surface since 2009 which supplies P to soil upon mineralization (Jat et al., 2017). With progress to crop growth stages, P concentration in soil decreased due to crop uptake; higher crop uptake in CSA based systems might be due to higher availability which is also supported by higher C mineralization in soil in those crop growth stages (Fig. 2). In case of K, luxury consumption under CSA based practices takes place leading to higher uptake.

### 4.5 Dry matter accumulation (DMA) and grain yield

The DMA accumulation was increased with the progressing crop growth stages. Higher DMA with Sc3 and Sc4 was due to the range of improved CSA based management practices acting alone or in combination (Kakraliya et al., 2018). Avoiding puddling for rice in Sc3 and Sc4 together with full residue retention resulted higher DMA due to improved soil physical and chemical properties in comparison to Sc1 (Jat et al., 2015, 2017). Higher yield with CSA based scenarios attributed to higher DMA, higher nutrient availability, and improved soil properties (Jat et al., 2017; Kumar et al., 2018).

## 5 Conclusion

This study concludes that residue retention and zero tillage (ZT) under climate smart agriculture (CSA) based practices significantly enhance enzyme activity, nutrient availability and uptake at different growth stages of wheat as compared to conventional tillage (based management practices. Higher dry matter accumulation and crop yield were also associated with CSA practices. The information on biological activities at different growth stages of wheat will be very much helpful in modifying the existing input management practices for higher nutrient availability to plants as well as sustained crop productivity. Therefore, climate smart agriculture based crop management practices should be recommended and popularized among the farmers.

## Declaration of Competing Interest

The authors declare that the research was conducted in the absence of any commercial or financial relationships that could be construed as a potential conflict of interest.
